# Cancer chemotherapy: early experience with combined chemotherapy for HIV-infected Kaposi's sarcoma patients at Lighthouse clinic, Lilongwe, Malawi

**DOI:** 10.1186/1758-2652-13-S4-P216

**Published:** 2010-11-08

**Authors:** HF Mulinde, H Tweya, J Chiwoko, C Feldacker, S Phiri, M Nyirenda, R Weigel, L Mlundira

**Affiliations:** 1 Lighthouse Trust, Clinic, Lilongwe, Malawi; 2 Lighthouse Trust, Clinic Monitoring and evaluation, Lilongwe, Malawi

## Background

Kaposi's sarcoma(KS) is the most common AIDS related malignancy in Malawi. National guidelines recommend chemotherapy with vincristine, along with antiretroviral treatment (ART). Effectiveness of vincristine monotherapy is limited, is considered palliative and interrupted supply contributes to poor outcomes of KS patients. Lighthouse, a major provider of HIV related services, started 38 patients on ART due to KS in quarter 1, 2010 and overall, 1195 patients ever started ART due to KS.

## Purpose of the study

To improve KS patient care,we introduced a combined treatment with vincristine(V), bleomycin(B) and doxorubicin(D), supplied by the central hospital pharmacy, developed standardized monitoring forms and trained providers in KS management.

## Methods

Clinicians stage KS patients' tumor severity (T0-good risk, T1-poor risk)and presence of systemic illness (SO-good risk, S1-poor risk) using a standardized clinical assessment form and record information about prior chemotherapy and ART. The form guides clinical decisions on regimen selection and accurate dosage. From initiation of chemotherapy, clinical officers examine patients and record lesion size at each subsequent visit. Nurses administer the treatment. Doses vary depending on clinical findings and body surface area. A specialist physician reviews patients with side effects.

## Results

Between 2nd June and 30th July 2010, 48 adult KS patients (35men) all of them with a prior history of vincristine monotherapy, started combined chemotherapy: 30(62%) received BV and 18(38%) received DBV. No patient developed side effects so far, and all of them are alive. Forty-six(96%) patients were already on ART at the time of chemotherapy initiation. 44 on d4T/3TC/NVP, one on d4T/3TC/EFV and one on AZT/3TC/TDF/LOP/r. All patients had severe KS manifestations (T1) with skin edema, pulmonary or gastrointestinal involvement, but only 5(10%) had systemic illnesses (S1), such as pneumonia, pulmonary TB or malaria requiring stabilization and co medication prior to chemotherapy. Bleomycin and vincristine were not available for 1 week.

## Conclusions

Combination chemotherapy for KS patients appears to be feasible in a resource poor public health clinic setting and may be helpful to improve outcomes of patients non responsive to standard vincristine monotherapy and ART. Ensuring a continuous supply of all chemotherapy agents is a priority.

